# Noble Metal-Free TiO_2_-Coated Carbon Nitride Layers for Enhanced Visible Light-Driven Photocatalysis

**DOI:** 10.3390/nano10040805

**Published:** 2020-04-23

**Authors:** Bo Zhang, Xiangfeng Peng, Zhao Wang

**Affiliations:** National Engineering Research Center of Industry Crystallization Technology, School of Chemical Engineering and Technology, Tianjin University, Tianjin 300072, China; yinyan@tjufe.edu.cn (B.Z.); tassadar528@eyou.com (X.P.)

**Keywords:** dielectric barrier discharge plasma, oxygen vacancy, g-C_3_N_4_/TiO_2_, photodegradation, H_2_ evolution

## Abstract

Composites of g-C_3_N_4_/TiO_2_ were one-step prepared using electron impact with dielectric barrier discharge (DBD) plasma as the electron source. Due to the low operation temperature, TiO_2_ by the plasma method shows higher specific surface area and smaller particle size than that prepared via conventional calcination. Most interestingly, electron impact produces more oxygen vacancy on TiO_2_, which facilitates the recombination and formation of heterostructure of g-C_3_N_4_/TiO_2_. The composites have higher light absorption capacity and lower charge recombination efficiency. g-C_3_N_4_/TiO_2_ by plasma can produce hydrogen at a rate of 219.9 μmol·g^−1^·h^−1^ and completely degrade Rhodamine B (20mg·L^−1^) in two hours. Its hydrogen production rates were 3 and 1.5 times higher than that by calcination and pure g-C_3_N_4_, respectively. Electron impact, ozone and oxygen radical also play key roles in plasma preparation. Plasma has unique advantages in metal oxides defect engineering and the preparation of heterostructured composites with prospective applications as photocatalysts for pollutant degradation and water splitting.

## 1. Introduction

Photocatalytic technology, a promising strategy for addressing energy shortages and environmental pollution, is important for the production of hydrogen via water splitting and the degradation of organic pollutants [1,2,3,4]. TiO_2_, discovered by Fujishima in 1972 [5], is the most widely studied and applied semiconductor photocatalyst and is non-toxic, stable, and cheap [6,7,8]. Many preparation methods of TiO_2_ and TiO_2_ composite have been used, such as the sol-gel method [9], solvothermal method [10], and chemical vapor deposition method [11] and so on. However, a green, simple, cheap and energy-efficient way for catalyst preparation is still necessary.

Non-thermal plasma, which has relatively low bulk temperature and extremely high electron temperature, has excellent advantages in preparing catalysts [12,13,14]. The catalyst can be prepared quickly without serious agglomeration due to the low temperature and high energy of plasma. On the other hand, the nucleation and crystallization process of the catalyst is very unique in plasma [13]. Furthermore, the catalysts prepared using the plasma method have small particles, strong interaction and specific structures [15,16]. However, TiO_2_ alone can only absorb ultraviolet light (only 4% of solar energy), even if the light absorption properties of TiO_2_ are improved. In addition, higher photo-generated charge recombination efficiency also affects its photocatalytic activity [17].

Strategies have been proposed to increase the photocatalytic activity of TiO_2_ under visible light. For example, compounding TiO_2_ with a narrow bandgap semiconductor catalyst can enhance its absorption of visible light and construct a special heterostructure [18,19]. Doping elements can reduce the TiO_2_ bandgap and increase its light absorption range [20,21]. Loading noble metals to TiO_2_ as the co-catalyst can act as its active site to enhance photocatalytic activity [22,23]. TiO_2_-coated carbon nitride layers as composites for enhanced photocatalytic activity is a more attractive approach. The g-C_3_N_4_ is widely used in the degradation of organic pollutants and water splitting by visible light irradiation due to its strong visible light response, high thermal resistance and chemical stability [24,25,26,27]. g-C_3_N_4_/TiO_2_ composites can not only transfer the photo-generated charge of g-C_3_N_4_ to TiO_2_ to increase its charge separation efficiency, but also reduce bandgap to increase its visible light absorption region [28,29,30]. Ma et al. got highly photocatalytic water splitting performance with g-C_3_N_4_/TiO_2_ composites by solvothermal method under visible light [10]. Papailias et al. utilized high temperature calcination to synthesize g-C_3_N_4_/TiO_2_ nanocomposites for NO*_x_* removal [31].

In this work, g-C_3_N_4_/TiO_2_ composites were prepared by using dielectric barrier discharge plasma, and their photocatalytic activities were evaluated by degrading RhB and hydrogen evolution under visible light irradiation. Due to the characteristics of the plasma preparation method, g-C_3_N_4_/TiO_2_ composites have many different properties compared to that by the traditional calcination method. Meanwhile, the mechanism of photocatalytic process and dielectric barrier discharge plasma preparation was proposed, respectively. It is predictable that the plasma method for catalysts preparation will be a very promising field.

## 2. Materials and Methods

### 2.1. Materials

Tetrabutyl titanate (TBT), melamine, absolute ethanol, rhodamine B (RhB), and triethanolamine were purchased from Shanghai Aladdin Biochemical Technology Co. (Shanghai, China). All chemicals were used directly.

#### Synthesis of TiO_2_ and g-C_3_N_4_/TiO_2_

g-C_3_N_4_ was dispersed by ultrasound in 20 mL of ethanol. We added 3ml TBT slowly into the as-prepared g-C_3_N_4_ suspension under adequate stirring. Then, the as-prepared hybrid suspensions were stand still for 24 h. The sample was filtered after 24 h. and drying at 80 °C. The samples were divided into two portions.

One portion was treated by DBD plasma to obtain g-C_3_N_4_/TiO_2_ composites according to the procedure reported in previous work [32,33]. Figure S1a shows the DBD device. The plasma was generated by the high voltage generator that can provide a sinusoidal waveform at 22 kHz with a voltage range of 0 to 30 kV. There are two quartz plates and a quartz ring between the two electrodes, and the sample was placed in the quartz ring. The gas atmosphere of the DBD reactor was air. The average power and average voltage of DBD during catalyst preparation were 200 W and 100 V, respectively. One-time plasma operation proceeded for 3 min to restrict the heat effect, followed by manually stirring to expose the untreated samples outside. The operation was repeated 20 times, until total plasma treatment time was 1 h. As shown in Figure S1b, the infrared (IR) image taken by the IR camera (Ircon, 100PHT, Everett, WA, USA) shown that the temperature of DBD plasma was below 106 °C. Finally, the obtained samples were denoted as TCN*X*-D (*X* = 10, 30, 50, 70, 90). *X* is equal to the weight ratio of g-C_3_N_4_ in the composites. D represents the samples prepared by DBD plasma. Figure S2 shows the schematic illustration of preparation of g-C_3_N_4_/TiO_2_ composite.

For comparison, another portion of the sample was calcined 450 °C at a rate of 5 °C/min for 2 h and designated as TCN50-C (50 wt% g-C_3_N_4_/TiO_2_).

The preparation method of pure TiO_2_ is the same as the above method. The samples obtained were denoted as TiO_2_-D and TiO_2_-C according to the different preparation methods.

### 2.2. Characterization

A Rigaku D/Max-2500 V/PC diffractometer with Cu Kα1 radiation (Cu Kα1 α = 0.154 nm, 40 kV, 40 mA, 8°·min^−1^, Rigaku, Tokyo, Japan) was used to analyze the crystal phase of samples. Fourier transform infrared (FTIR) spectra was analyzed by a Bruker Alpha FTIR-attenuated total reflection (ATR) instrument (Bruker, Karlsruhe, Germany). A Biaode SSA-7000 analyzer (Biaode Electronic Technology Ltd., Beijing, China) was applied to determine specific surface area and pore size. Thermogravimetry analysis was conducted on Perkin-Elmer TGA/DTA thermo-gravimetric analyzer (Waltham, MA, USA) in O_2_ atmosphere. The temperature range was 20–800 °C. An ULVAC-PHI-5000versaprobe instrument (Tokyo, Japan) was used to obtain X-ray photoelectron spectra. The adventitious carbon C1s used for element correction is located at 284.8 eV. Electron paramagnetic resonance measurement was obtained on a Bruker A300 spectrometer (Karlsruhe, Germany) at room temperature. The ultraviolet–visible (UV–vis) diffuse-reflectance spectroscopy was recorded by a UV-2600 spectrophotometer (Shimadzu, Kyoto, Japan). Photoluminescence (PL) spectroscopy was analyzed on Hitachi F-4600 spectrometer (Tokyo, Japan) with excitation at 350 nm. Scanning electron microscopy (SEM) and energy dispersive X-ray spectroscopy were conducted on Hitachi S4800 instrument (Tokyo, Japan). High-resolution transmission electron microscopy (HRTEM) was obtained using a JEM-2100F instrument (JEOL, Tokyo, Japan).

### 2.3. Measurement of Photocatalytic Activity

#### 2.3.1. RhB degradation

We mixed 50 mg samples and 100 mL RhB (20 mg·L^−1^) solution uniformly in continuous stirring. First the mixture was stirred without light for 0.5 h to obtain the balance of adsorption and desorption. Visible light was provided by Xenon lamp (HSX-F300, 300 W) with a 420 nm cut filter. The illumination power was 100 mW/cm^2^. The light source was placed about 10 cm above the RhB solution. In the experiment, 4 mL solution was taken every 20 min for RhB degradation rate test. The degradation rate of the obtained RhB solution was determined using a UV-2600 instrument.

#### 2.3.2. Hydrogen Generation

The H_2_ evolution experiment was conducted on the PerfectLight 3AG instrument (Beijing, China). The amount of H_2_ was determined by online gas chromatography. 100 mg sample was added to 70 mL of solution containing 10% triethanolamine and 133 μL of H_2_PtCl_6_ (0.5 wt%). The mixed solution was placed in 100 mL closed glass container. The 300 W xenon lamp was equipped with total reflection and 420 nm filter to provide full-spectrum light and visible light, respectively. The light source was placed 10 cm above the suspension. After full-spectrum irradiation for 3 h in vacuum, the Pt was loaded on the samples. Then the hydrogen production reaction started, and the content of H_2_ in the system was measured by online gas chromatography per hour.

#### 2.3.3. Photoelectrochemical Investigation

The photocurrent density was conducted at the LK2010 electrochemical system. It is a traditional three-electrode system, tin oxide mixed with fluorine (FTO) conductive glass loaded with sample as working electrode. The working electrode was made as follows: 50 mg of the sample and 10 μL of nafion (5%) were added to 500 μL absolute ethanol, 200 μL of the suspension was loaded on FTO glass after mixing evenly. Then the FTO working electrode was obtained by drying at room temperature for 12 h. The reference electrode and counter electrode were the saturated Hg/HgO electrode and Pt wire, respectively. The 0.1M Na_2_SO_4_ solution was used as electrolyte. Visible light was provided by 300 W Xe lamp equipped with 420 nm filter. At the beginning of the test, we fully introduced N_2_ into the electrolyte to remove dissolved oxygen. The experimental potential is 0.4 eV, which is the optimal value by testing (see Figure S3).

## 3. Results and Discussion

### 3.1. Physico-Chemical Properties

**Figure 1:**
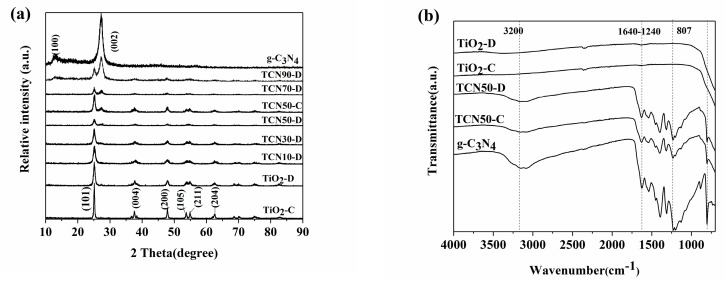
(**a**) XRD patterns of TiO_2_, g-C_3_N_4_ and TCN*X* samples, (**b**) FTIR spectra of TiO_2_, g-C_3_N_4_, TCN50 samples.

The crystal phases of g-C_3_N_4_, TiO_2_, and g-C_3_N_4_/TiO_2_ composites were analyzed by using wide-angle X-ray diffraction (XRD). As shown in Figure 1a, g-C_3_N_4_ exhibits (100) and (002) planes at 13.2° and 27.6°, which correspond to tri-s-triazine structure and stacking of the conjugated aromatic system, respectively [34]. The as-prepared TiO_2_ present the diffraction peaks at 25.1°, 37.7°, 47.8°, 54.0°, 55.1° and 62.5°, which corresponded to the (101), (004), (200), (105), (211) and (204) crystal planes of anatase TiO_2_ (JCPDS 71-1166), respectively [35]. It indicated TiO_2_ was successfully prepared by DBD plasma. As the proportion of g-C_3_N_4_ increased in g-C_3_N_4_/TiO_2_ composites, the intensities of g-C_3_N_4_ peaks increased gradually. However, the no peak position shift of TiO_2_ indicates that g-C_3_N_4_ has no influence on the crystal structure of TiO_2_. Moreover, compared with samples (TiO_2_ and g-C_3_N_4_/TiO_2_ composites) prepared by high-temperature calcination, the samples by DBD plasma have lower peak intensity and wider peak width. On the basis of the full width at half maximum (FWHM) of (101) crystal plane and Debye–Scherrer equation, the average crystalline sizes of TiO_2_ in the g-C_3_N_4_/TiO_2_ composites were calculated and are listed in Table 1 [36]. The crystalline sizes of TiO_2_ in the composites decreased gradually as g-C_3_N_4_ increased. It is worth noting that TiO_2_ has smaller crystallite size than that by the calcination method. The relative low temperature of plasma is the main reason restricting the agglomeration of particles.

Fourier transform infrared spectra (FTIR) of g-C_3_N_4_, TiO_2_, and g-C_3_N_4_/TiO_2_ composites are shown in Figure 1b. It reveals the composition and chemical bonding of samples. For pure g-C_3_N_4_, the peak of 807 cm^−1^ is due to the tri-s-triazine unit structure, and four intense bands in the region 1240–1640 cm^−1^ are attributed to the stretching of the C–N heterocycle in g-C_3_N_4_ [37]. For TiO_2_-C and TiO_2_-D, they showed similar peaks, and the shoulder bands between 3000–3500 cm^−1^ can be attributed to –OH stretching vibration [38]. The shoulder bands near 3200 cm^−1^ in TCN50 composites are contributed by the N-H stretching vibration modes [37,39]. TCN50 composite exhibited both the g-C_3_N_4_ and TiO_2_ characteristic peaks. This shows that g-C_3_N_4_/TiO_2_ composites were directly prepared by DBD plasma and is agreement with XRD analysis results. During plasma process, high energy electron bombard samples. It can be deduced that the surface hydroxyl and amino groups enhance interaction between TiO_2_ and g-C_3_N_4_ [40]. The strong interfacial connection can be used as the channel for charge conduction to improve charge separation efficiency.

Nitrogen adsorption–desorption isotherms and pore-size distribution curves of g-C_3_N_4_, TiO_2_ and TCN50 samples are shown in Figure S4. It is clearly observed that samples prepared by plasma have larger N_2_ adsorption capacity. The specific data are listed in Table 1. The specific surface area of TiO_2_-D (64.56 m^2^/g) is approximately 2.3 times that of TiO_2_-C (28.18 m^2^/g), which can be ascribed to the low agglomeration of the sample’s particles prepared by low-temperature plasma. The specific surface area of g-C_3_N_4_ merely is 7.13 m^2^/g. As shown in Table S1, the specific surface area and pore volume of g-C_3_N_4_/TiO_2_ composites decreased and the average pore radius increased gradually as the proportion of g-C_3_N_4_ increased. Compared with TCN50-C, TCN50-D has higher specific surface area, pore volume, and smaller average pore radius, which indicates that the surface of the plasma-treated sample can expose more active sites. In addition to the low temperature, the repulsion between the electrons attached to the particles prevents the agglomerating of particles [12], resulting in the excellent dispersion of TiO_2_ on g-C_3_N_4_ to enhance photocatalytic activity.

Thermogravimetry (TG) was used to analyze thermal stability of the samples. As shown in Figure S5, the weight of TiO_2_ prepared by DBD plasma only reduced by 2%, which indicated that DBD plasma can complete decompose TBT to TiO_2_ at moderate temperature. The g-C_3_N_4_ gradually lost weight from 550 °C to 730 °C. The composites TCN*X* gradually lost weight from 520 °C to 650 °C, mainly due to the burning of g-C_3_N_4_. The thermal stability of g-C_3_N_4_ was reduced after coating with TiO_2_. The reason is catalytic action of TiO_2_ and the cross-linking ring of g-C_3_N_4_ after compounding [41]. Ignoring the slight weight loss due to water, the actual proportions of g-C_3_N_4_ in the composites TCN*X*-D (*X* = 10, 30, 50, 50, 70, 90) were 13.0, 30.3, 52.9, 71.3, and 90.0 wt%, respectively. The proportion of g-C_3_N_4_ in TCN50-C was 53.6%, which was approximately equal to the ideal ratio of TCN50-D.

### 3.2. Characterizations of Oxygen Vacancies

Chemical states of elements in g-C_3_N_4_, TiO_2_, and g-C_3_N_4_/TiO_2_ composites were investigated by X-ray photoelectron spectroscopy (XPS). Figure S6a shows the high-resolution C 1s spectrum of the prepared samples. All samples have two C 1s peaks at 284.8 and 288.3 eV, which can be ascribed to the inherent adventitious carbon and N-C-N coordination, respectively [42]. Compared with the N-C-N peak of pure g-C_3_N_4_, TCN50 exhibit weaker peak intensity and positive shifts of 0.4 eV in binding energies, which indicates there is a chemical interaction between TiO_2_ and g-C_3_N_4_, hence, g-C_3_N_4_ has close surface contact with TiO_2_ [43]. As shown in Figure S5b, for the N 1 s high-resolution spectrum of pure g-C_3_N_4_ and g-C_3_N_4_/TiO_2_ composites, three peaks were observed at about 398.8, 399.6, and 401.2 eV. The first peak is attributed to sp2 hybridized nitrogen (C=N–C), the second peak is due to the tertiary N in N–(C)^3^ groups, and the last peak corresponds to the existence of amino groups (C–N–H) [44,45].

**Figure 2:**
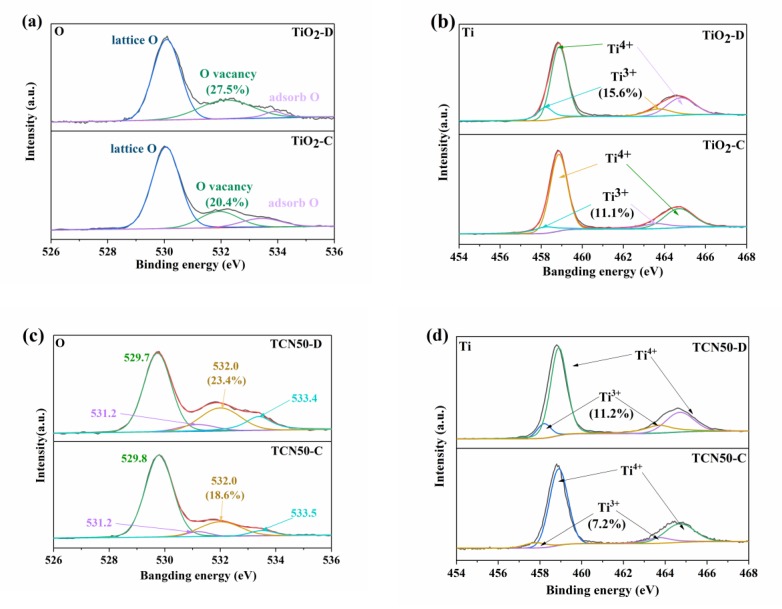
The high-resolution XPS spectra: (**a**) O 1s, (**b**) Ti 2p, of TiO_2_ samples. (**c**) O 1s, (**d**) Ti 2p of TCN50 samples.

Figure 2a,b shows O 1s and Ti 2p spectrum region of pure TiO_2_. Figure 2a shows the O 1s spectrum region of TiO_2_-D have three peaks at about 529.7, 532.0 and 533.4 eV, which corresponds to the lattice oxygen, the oxygen vacancy and the adsorbed oxygen, respectively [46,47]. By calculating the ratio of the area occupied by oxygen vacancies, it was 23.4% and 18.6% in TiO_2_-D and TiO_2_-C, respectively, indicating that plasma method can produce more oxygen vacancies than that of the calcination method in TiO_2_. Figure 2b shows the Ti 2p spectrum of pure TiO_2_, There are four peaks at about 458.2, 463.7, 458.9, and 464.6 eV, corresponding to Ti^3+^ 2p_3/2_, Ti^3+^ 2p_1/2_, Ti^4+^ 2p_3/2_ and Ti^4+^ 2p_1/2_, respectively [46,47]. It was found that the area of Ti^3+^ in TiO_2_-D and TiO_2_-C were 15.6% and 11.1%, respectively. Compared with TiO_2_ by calcination, the valence of more Ti in TiO_2_ by plasma is reduced from Ti^4+^ to Ti^3+^. This is consistent with the observation for the oxygen vacancy. During the plasma preparation of TiO_2_, the oxygen atoms escaped to form oxygen vacancies and trivalent Ti [46].

Figure 2c,d shows O 1s and Ti 2p spectrum of TCN50 composites. TCN50 and TiO_2_ show similar peaks at O 1s and Ti 2p. However, the intensity of the peaks representing oxygen vacancies and Ti^3+^ decreased. The ratio of oxygen vacancies and Ti^3+^ were 23.4% and 11.2% in TCN50-D, 18.6% and 7.2% in TCN50-C, respectively. It is indicated that g-C_3_N_4_ occupies the oxygen vacancies of TiO_2_ after TiO_2_ coated on g-C_3_N_4_. This will undoubtedly strengthen the interaction of TiO_2_ and g-C_3_N_4_. It is in agreement with previous results.

**Figure 3:**
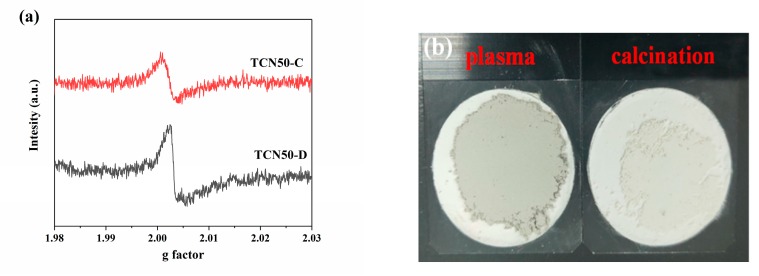
(**a**) EPR spectra of TCN50-C and TCN50-D samples, (**b**) images of TiO_2_ prepared by plasma and calcination.

To further verify the existence of oxygen vacancies, electron paramagnetic resonance (EPR) analysis of the TCN50 composites was performed. As shown in Figure 3a, both TCN50-C and TCN50-D have the EPR signal at *g* = 2.003, which means the appearance of oxygen vacancies [48]. The peak intensity of TCN50-D is stronger than TCN50-C, indicating the plasma method produces more oxygen vacancies. As can be seen from Figure S7, the EPR spectra of TiO_2_-C and D exhibit the same characteristics as that of TCN50. This is consistent with XPS results. In addition, Figure 3b shows different colors between TiO_2_-D and TiO_2_-C. The color of TiO_2_-C is white, while TiO_2_-D is gray. This indicates oxygen vacancies can narrow band gap to promote light harvesting, leading significant color change [49].

### 3.3. Optical Properties

**Figure 4:**
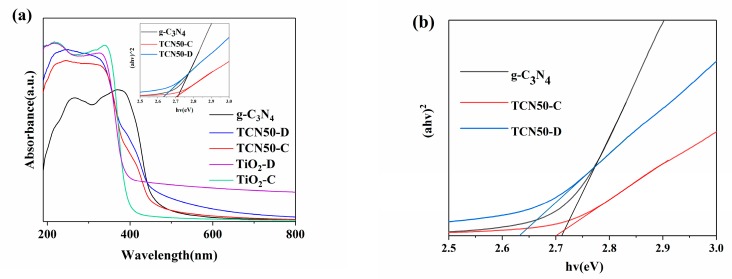

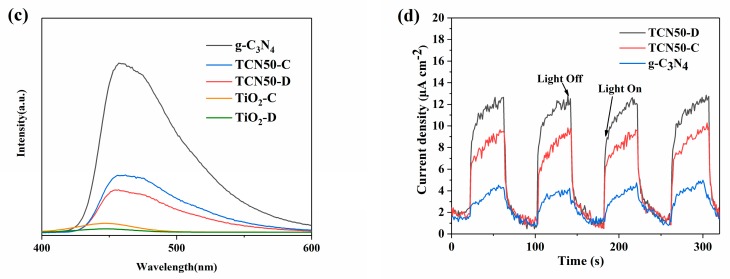
(**a**) UV–vis DRS of TiO_2_, g-C_3_N_4_, TCN50-C and TCN50-D samples. (**b**) The relationship between (ahv)^2^ and photo energy. (**c**) Photoluminescence spectra of g-C_3_N_4_, TiO_2_ and TCN50 samples. (**d**) Photocurrent density vs. time for g-C_3_N_4_/FTO, TCN50-C/FTO and TCN50-D/FTO.

Ultraviolet–visible diffuse reflection spectrum (UV-vis DRS) is shown in Figure 4a and Figure S8 reveal the light absorption capacity and bandgaps of the as-prepared g-C_3_N_4_, TiO_2_, and TCNX. The critical values of the light response of TiO_2_-D and TiO_2_-C were found at 398 nm and 388 nm, indicating that bandgaps were 3.12 and 3.19 eV, respectively [50]. The absorption starting point of g-C_3_N_4_ was located at 458 nm. The absorption range of visible light of TCN50 composites was significantly expanded due to introducing g-C_3_N_4_. As shown in Figure 4b, on the basis of the Kubelka-Munk formula, the bandgaps of g-C_3_N_4_, TCN50-C, and TCN50-D were calculated to be 2.71, 2.70, and 2.63 eV, respectively. Based on the above results, the samples prepared by plasma had a narrower bandgap. This can be attributed to oxygen vacancies [51]. Oxygen vacancies can introduce a defect status below the conduction band, and thus narrows the bandgap to improve the light absorption range [46,52]. Hence, TCN50-D has a relatively strong visible light response capacity.

Photoluminescence (PL) spectra was shown in Figure 4c. The PL peak intensity is proportional to the recombination rates of the photogenerated electron-hole pairs [53]. g-C_3_N_4_ has strong peak intensity, indicating that its charge separation efficiency is low. TiO_2_-D has weaker PL peak strength than that of TiO_2_-C, indicating that TiO_2_-D is more conducive to charge transport. After TiO_2_ coated on g-C_3_N_4_, PL peak intensity was greatly reduced, because electrons can be transmitted through the interface of g-C_3_N_4_ and TiO_2_ [54]. TCN50-D shows lower peak intensity than TCN50-C, resulting in that TCN50-D has lower charge recombination rate and stronger photocatalytic activity. Meanwhile, the presence of oxygen vacancies can promote the separation of electrons and holes [55]. The oxygen vacancies not only restrain the recombination of charges, but also narrow the band gap to increase light absorption [52,56]. As shown in Figure 4d, TCN50-D exhibits the highest photocurrent density than TCN50-C, indicating that TCN50-D has higher photoelectric conversion efficiency [57].

### 3.4. Morphologies

**Figure 5:**
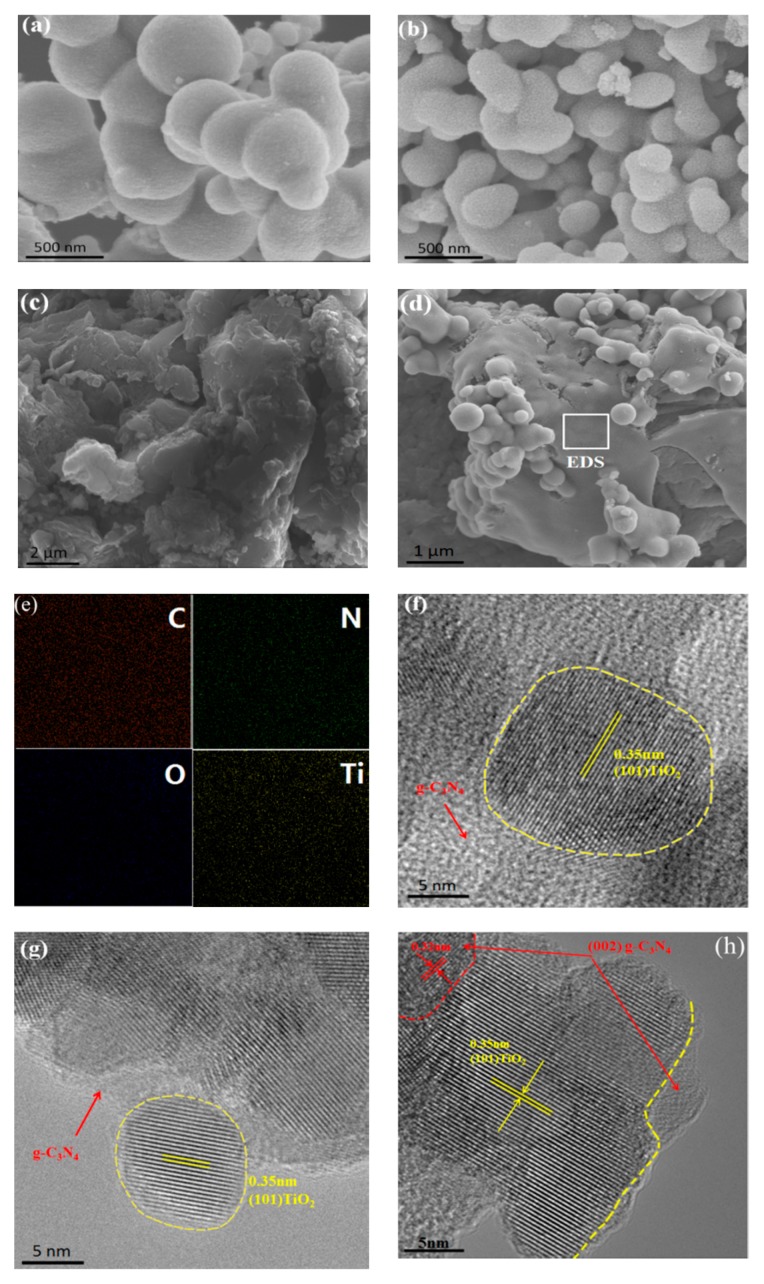
SEM images of (**a**) TiO_2_-C, (**b**) TiO_2_-D, (**c**) g-C_3_N_4_, (**d**) TCN50-D and (**e**) EDS elemental mappings of TCN50-D samples; high-resolution transmission electron microscopy (HRTEM) images of (**f**) TCN50-C sample, (**g**,**h**) TCN50-D sample.

Figure 5 shows the morphology and microstructure of TiO_2_, g-C_3_N_4_ and g-C_3_N_4_/TiO_2_ composites. As shown in Figure 5a,b, TiO_2_-D has less particle aggregation than TiO_2_-C, which agreed well with previous result. As shown in Figure 5c,d, g-C_3_N_4_ has an anomalous layered structure and smooth flat surface. After TiO_2_ coating process by plasma, TiO_2_ was uniformly dispersed on the surface of g-C_3_N_4_, and the surface of g-C_3_N_4_ changes from smooth to rough. Figure 5e and Figure S9 show the energy dispersive X-ray spectroscopy (EDS) mapping of TCN50-D, the four elements (C, N, O, Ti) are well-dispersed in the TCN50-D composite. It can form a heterostructure for electron transport and reduce charge recombination efficiency.

To further investigate the microstructure of g-C_3_N_4_/TiO_2_ composites, HRTEM was conducted. As can be seen from Figure 5f,g, the particle size of TiO_2_ in TCN50-C composite is around 18 nm, that in the TCN50-D composite is around 12 nm. This proves that plasma treated sample has smaller particle size. The lattice spacing of TiO_2_ is 0.35 nm representing the (101) plane of anatase titanium oxide. Figure 5h also shows the clear lattice fringe of g-C_3_N_4_ is 0.32 nm, corresponding to the (002) lattice plane of g-C_3_N_4_ [54]. Compared with TCN50-C composite, TCN50-D have more and tighter heterojunctions, which also means extensive interfacial contacts to enhance photocatalytic activity.

### 3.5. Photocatalytic Activity

**Figure 6:**
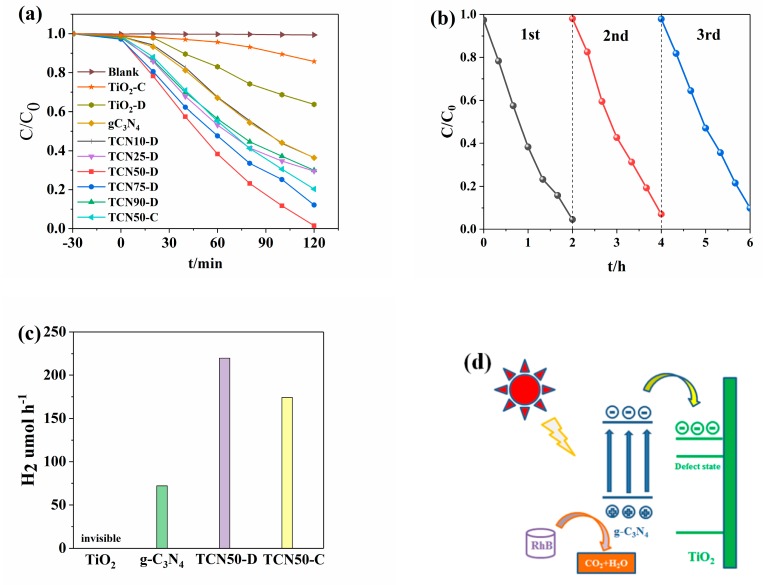
(**a**) Photocatalytic degradation of RhB under visible light irradiation. (**b**) The photodegradation stability of RhB over TCN50-D sample. (**c**) Photocatalytic H_2_ evolution rates of TiO_2_, g-C_3_N_4_, TCN50-C and TCN50-D under visible light irradiation. (**d**) Photocatalytic mechanism for the charge transfer between g-C_3_N_4_ and TiO_2_ under visible light irradiation.

RhB degradation and H_2_ production under visible light were conducted to evaluate the photocatalytic performance of the samples. As shown in Figure 6a, the concentration of RhB hardly changed within 0.5 h of dark adsorption. For TiO_2_-C and TiO_2_-D, RhB was degraded by 14.3% and 33.7% under two hours of the irradiation of visible light, respectively, which can be ascribed to the sensitization of dye [58]. Plasma-treated TiO_2_ show better photoactivity. Pure g-C_3_N_4_ degraded 63.7% of RhB under two hours of visible light irradiation. With the increase of proportion of g-C_3_N_4_ in g-C_3_N_4_/TiO_2_, the degradation efficiency increased first and then decreased; 50 wt% g-C_3_N_4_ in g-C_3_N_4_/TiO_2_ composite exhibited the highest RhB degradation efficiency. When the amount of g-C_3_N_4_ is low, TiO_2_ covers its surface and prevents absorption of visible light. However, when the amount of TiO_2_ is low, it is insufficient to promote charge separation. Therefore, the appropriate ratio of g-C_3_N_4_/TiO_2_ has higher photocatalytic activity. Compared with 79.7% RhB degradation efficiency of TCN50-C, TCN50-D can completely degrade RhB in two hours, due to its stronger visible light absorption and charge separation efficiency.

Furthermore, Figure 6b shows the reusability experiment of the TCN50-D sample. The photocatalytic activity for degradation of RhB decreased from 95.5% to 90.2% within 6 h under visible light after the third cycle. This indicated that the g-C_3_N_4_/TiO_2_ composite by plasma has high stability.

As shown in Figure 6c, the hydrogen production of pure TiO_2_ was not detected in the system. Pure g-C_3_N_4_ only produced 72.1 μmol·g^−1^ H_2_ per hour. The hydrogen production of TCN50-D and TCN50-C composites was 219.9 and 174.3 μmol·g^−1^·h^−1^, respectively. TCN50-D exhibited higher photocatalytic activity and approximately 3 times that of pure g-C_3_N_4_ and 1.26 times that of TCN50-C.

### 3.6. Mechanism

In order to investigate the phtotcatalytic degradation mechanism of the TCN50-D composite, different scavengers were added before RhB degraded by TCN50-D. As shown in the Figure S10, the degradation activity of TCN50-D changed slightly after the addition of the hydroxyl radical scavenger isopropyl alcohol (IPA) and the superoxide radical scavenger p-benzoquinone (BQ). However, after the addition of the hole scavenger Ethylene Diamine Tetraacetic Acid-2Na (EDTA-2Na), a significant decrease in photocatalytic activity for RhB degradation was observed, indicating that the hole is the main active species for degrading RhB. By measuring the intersection of the slope of the XPS valence band curve and the X axis, the valence band (VB) of the samples can be determined. As shown in Figure S11, the VB of TCN50-C and TCN50-D is 2.32 eV and 2.38 eV, respectively, indicating that TCN50-D possesses stronger oxidizing ability [40]. The VB of TiO_2_-D and TiO_2_-C is 2.91 eV and 2.84 eV, respectively. The conduction band (CB) of TiO_2_-D can be calculated that is −0.20 eV, according to its band gap of 3.11 eV. The VB and CB of g-C_3_N_4_ are 2.24 eV and −0.47 eV, respectively [40]. Therefore, the energy band structure model of TCN50-D can be constructed and shown in Figure 6d. Under visible light irradiation, g-C_3_N_4_ captures photons to generate electron-hole pairs, then electrons can be transported from the CB of g-C_3_N_4_ to the CB of TiO_2_-D to reduce H^+^ to produce H_2_. Meanwhile, the holes in the VB of g-C_3_N_4_ can oxidize RhB to exert photodegradation [59].

The mechanism of TiO_2_ and g-C_3_N_4_/TiO_2_ composite prepared by dielectric barrier discharge plasma is proposed as follows. The high-energy electron bombardment plays a key role in the process.

During the plasma process, there are a large number of electrons that can decompose titanium hydroxide through breaking bonds by electron bombardment (Equation (1)). This also can produce oxygen vacancies.

Ti(OH)_4_ + e → TiO_2_ + H_2_O (1)

Under the influence of plasma, the ozone and oxygen atoms can be generated by using Equations (2)–(4) [16,60].

e + O_2_ → e + O(^3^P) + O(^3^P) (2)

e + O_2_ → e + O(^1^D) + O(^3^P) (3)

O + O_2_ + M → O^3^* +M → O^3^ + M (4)

As shown in Equations (5) and (6), the oxygen atom and ozone can also lead to Ti(OH)_4_ decomposition.

Ti(OH)_4_ + O → TiO_2_ + 2H_2_O + 1/2O_2_ (5)

Ti(OH)_4_ + O_3_ → TiO_2_ + 2H_2_O + 3/2O_2_ (6)

## 4. Conclusions

TiO_2_ and g-C_3_N_4_/TiO_2_ composites were prepared by DBD plasma, which is a green, easy, and efficient method for catalyst preparation. Here, TiO_2_ by plasma has enriched oxygen vacancies and larger specific surface area due to the electron impact. The electron impact, ozone and oxygen radical play important role in plasma preparation, which facilitate the interaction of TiO_2_ and g-C_3_N_4_ and forms heterojunctions. g-C_3_N_4_/TiO_2_ composites by plasma have stronger light absorption capacity and higher charge separation efficiency. TCN50-D exhibited the highest photocatalysis activity on RhB degradation and hydrogen production, which was ascribed to enriched oxygen vacancies and special heterostructures. Plasma, green and convenient technology, provides a promising strategy for oxide defect engineering and composite preparation.

## Figures and Tables

**Table 1:** Surface and structural characterization of TiO_2_, g-C_3_N_4_ and TCN*X* composites.

Sample	S_BET_ (m^2^/g)	Pore Volume (cm^3^/g)	Average Pore Radius (nm)	Crystallite Size (nm)
TiO_2_-D	64.5649	0.1566	2.99	14.3
TiO_2_-C	28.1876	0.1023	6.79	17.8
TCN50-D	72.8473	0.1451	2.86	12.3
TCN50-C	29.2735	0.1117	6.02	14.4
g-C_3_N_4_	7.1313	0.0604	11.34	-

## Supplementary Materials

The following are available online at https://www.mdpi.com/2079-4991/10/4/805/s1: Figure S1: The dielectric barrier discharge (DBD) plasma reactor and infrared (IR) image of DBD plasma, Figure S2: The schematic illustration for the preparation of TCN composites photocatalysts, Figure S3: Photocurrent tests of TCN50-D at different potential, Figure S4: Nitrogen adsorption–desorption isotherms (a) and pore-size distribution curves (b) of g-C_3_N_4_, TiO_2_ and TCN50 samples, Figure S5: Thermogravimetric analysis curves, Figure S6: The high-resolution XPS spectra C 1s and N 1s, Figure S7: The EPR spectra of TiO_2_-C and D samples, Figure S8: FTIR spectra, UV–vis DRS and XPS valence band (VB) spectra of TCNX, Figure S9: EDS patterns and element mapping of TCN50-D sample, Figure S10: Trapping experimental of photogenerated radicals and holes in TCN50-D sample for the RhB degradation, Figure S11: VB XPS of TiO_2_-C, TiO_2_-D, TCN50-C and TCN50-D, Table S1: Surface and structural characterization of TiO_2_, g-C_3_N_4_ and TCNX composites.
