# Supplementary material for: Noble Metal-Free TiO2-Coated Carbon Nitride Layers for Enhanced Visible Light-Driven Photocatalysis
Source: Nanomaterials (Basel). 2020 Apr 23;10(4):805. doi: 10.3390/nano10040805 (PMC7221541; doi:10.3390/nano10040805)
Supplement: Supplementary file 1 [file nanomaterials-10-00805-s001.pdf]

Supplementary Material

Noble-metal-free TiO_2 coated carbon nitride layers for enhanced visible-light-driven photocatalysis

Bo Zhang, Xiangfeng Peng, Zhao Wang*

National Engineering Research Center of Industry Crystallization Technology, School of Chemical Engineering and Technology, Tianjin University, Tianjin 300072, China

Corresponding authors: wangzhao@tju.edu.cn

S1. Synthesis of g-C_3N_4

g-C_3N_4 powder was synthesized according to a classic procedure of Ma et al [1]. The pure g-C_3N_4 powder was synthesized by heating melamine powder directly. Typically, 10 g melamine was putted into a closed alumina crucible, then heated at 550℃ for 4 h with the rate of 5℃/min in the muffle furnace. The as-prepared yellow product was grounded for further use.

S2. The DBD plasma reactor and IR image of DBD plasma.

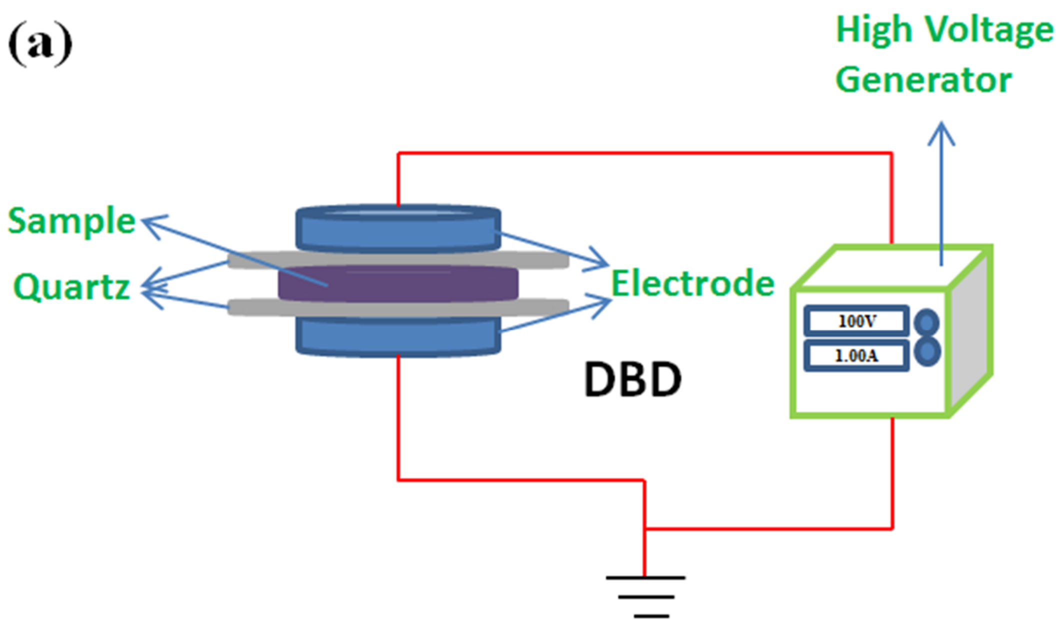

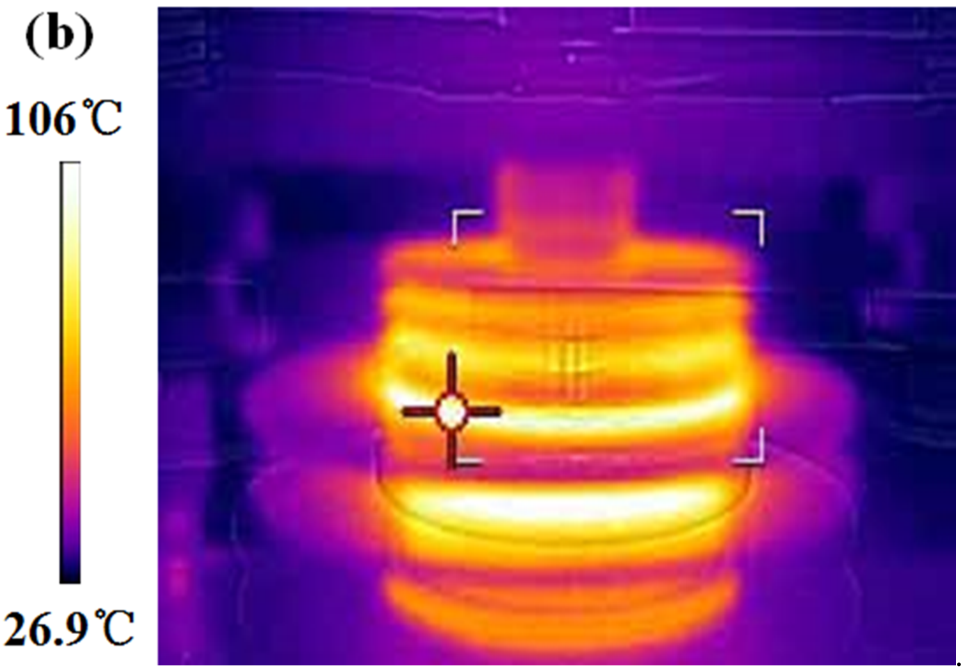

Figure S1. (a) The DBD plasma reactor and (b) IR image of DBD plasma reactor during samples preparation.

S3. The schematic illustration for the preparation of TCN composites photocatalysts.

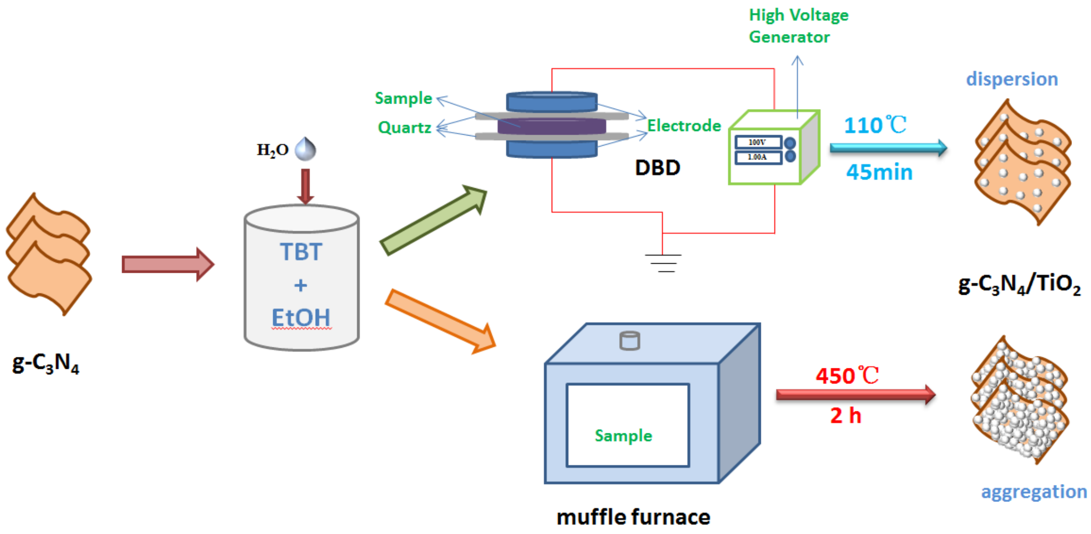

Figure S2. The schematic illustration for the preparation of TCN composites photocatalysts

S4. Photocurrent tests of TCN50-D at different potentials.

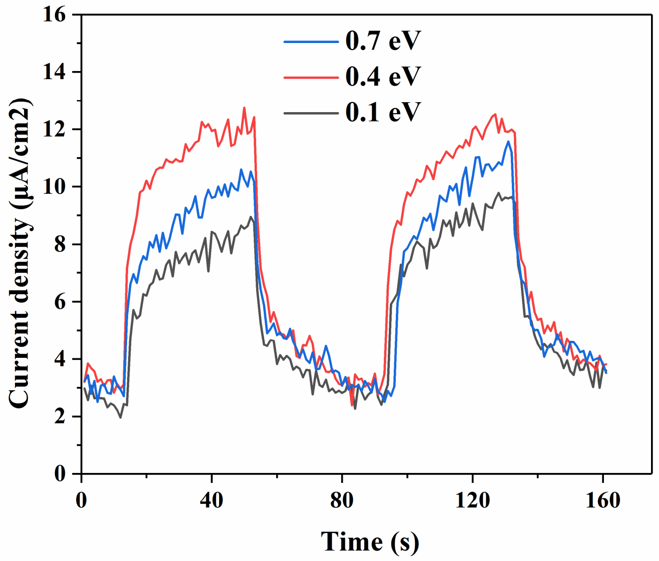

Figure S3. Photocurrent tests of TCN50-D at different potential.

S5. Nitrogen adsorption–desorption isotherms.

Table S1. Surface and structural characterization of TiO_2, g-C_3N_4 and TCNX composites.

Sample	S_{BET} (m²/g)	Pore volume(cm³/g)	Average pore radius(nm)	Crystallite size (nm)
TiO_2-D	64.6±6.1	0.16±0.02	2.99±0.3	14.3
TiO_2-C	28.2±2.4	0.10±0.01	6.79±0.6	17.8
**TCN10-D**	117.2±10.3	0.17±0.02	2.29±0.2	13.7
**TCN30-D**	94.9±8.8	0.16±0.02	2.80±0.3	13.4
**TCN50-D**	72.8±6.3	0.14±0.01	2.86±0.3	12.3
**TCN50-C**	29.3±2.7	0.11±0.01	6.02±0.6	14.4
**TCN70-D**	43.4±4.0	0.12±0.01	4.04±0.4	11.4
**TCN90-D**	35.6±3.1	0.11±0.01	5.38±0.5	10.8
g-C_3N_4	7.1±0.9	0.06±0.01	11.34±1.0	/

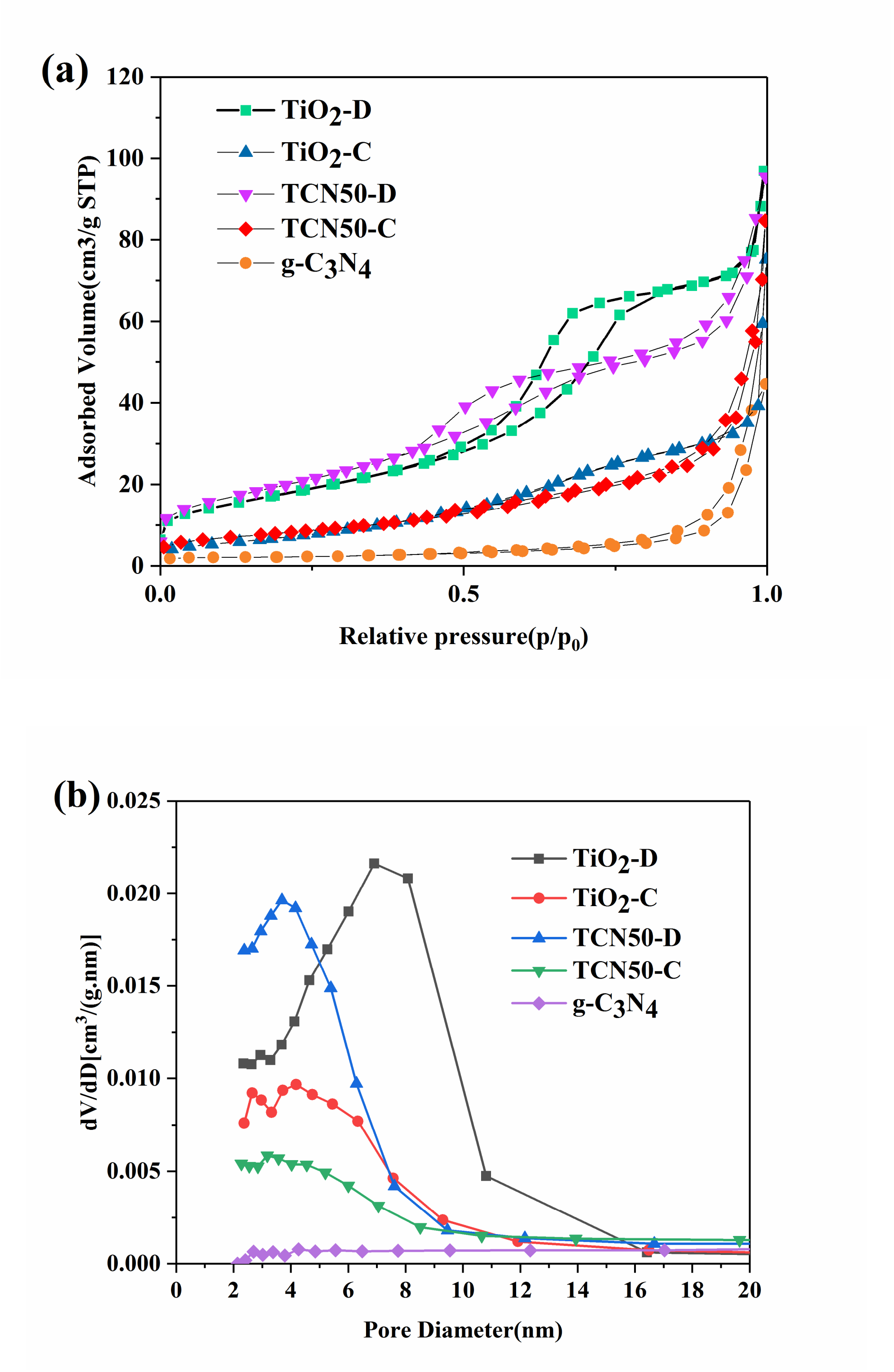

Figure S4. Nitrogen adsorption–desorption isotherms (a) and pore-size distribution curves (b) of $g-C_3N_4$, TiO_2 and TCN50 samples.

S6. Thermogravimetric Analysis curves

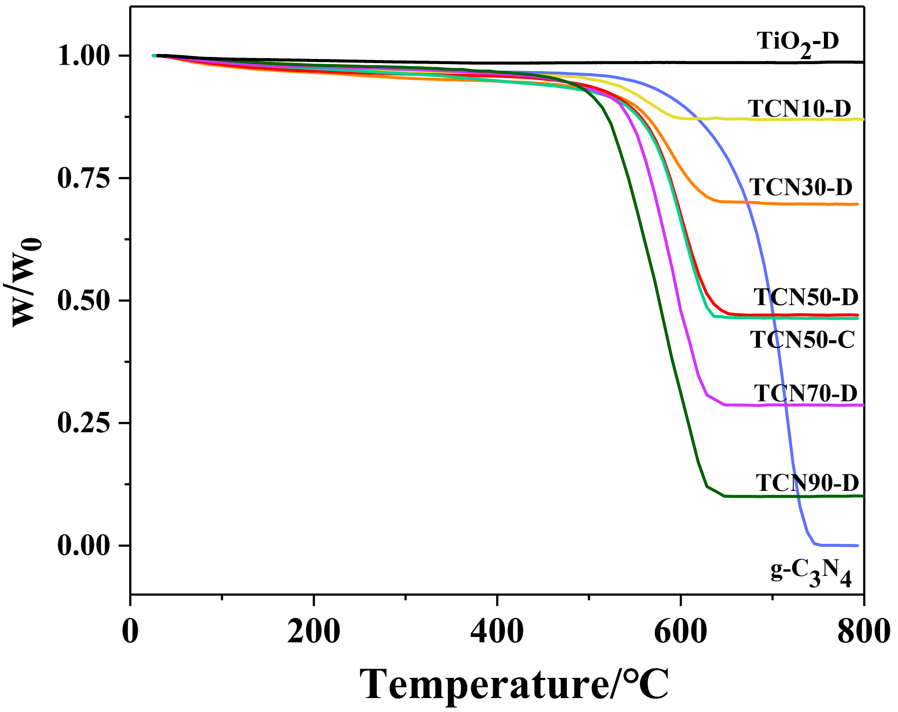

Figure S5. TGA curves of the g-C_3N_4, TiO_2-D and TCNX composite photocatalysts.

S7. The high-resolution XPS spectra C 1s and N 1s

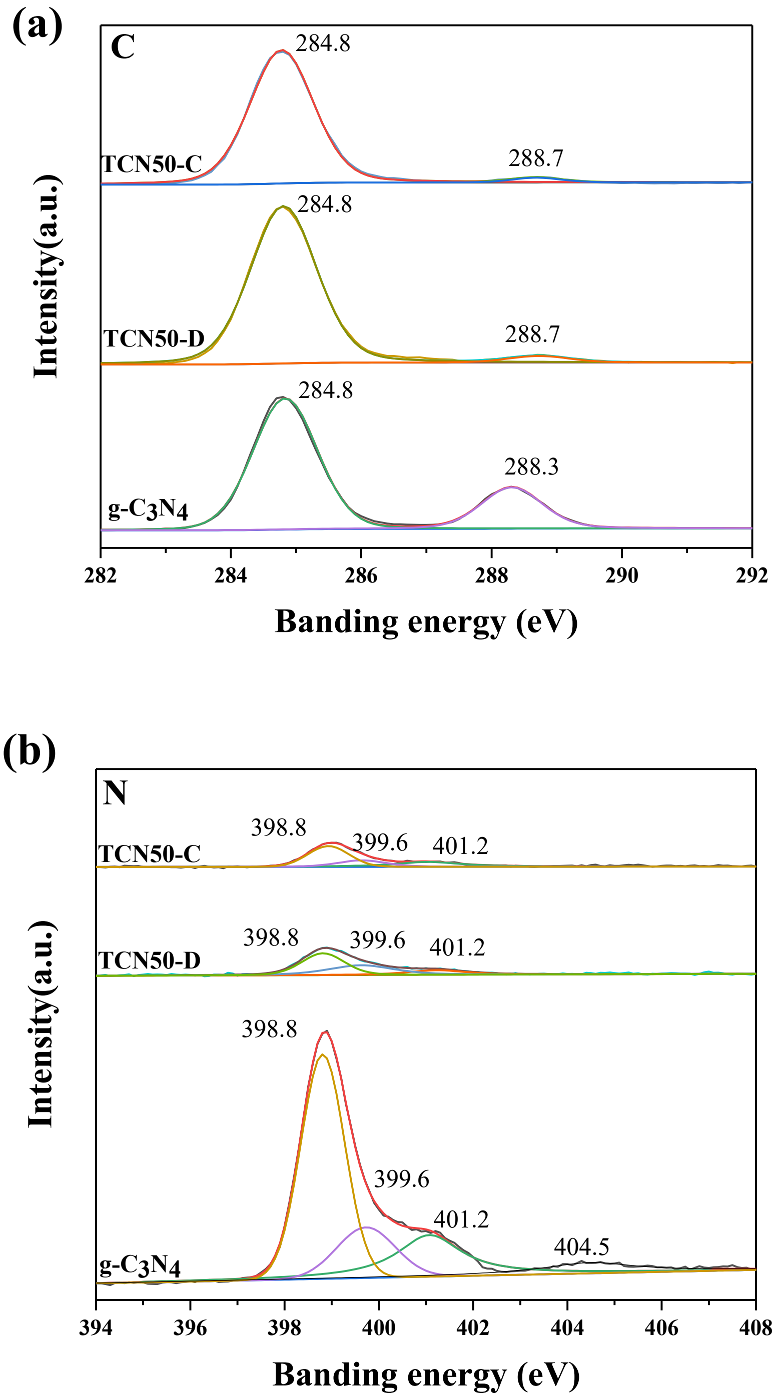

Figure S6. (a) The high-resolution XPS spectra C 1s, (b) N 1s of g-C_3N_4, TCN50-C and TCN50-D samples.

S8. The EPR spectra of TiO_2-C and D samples

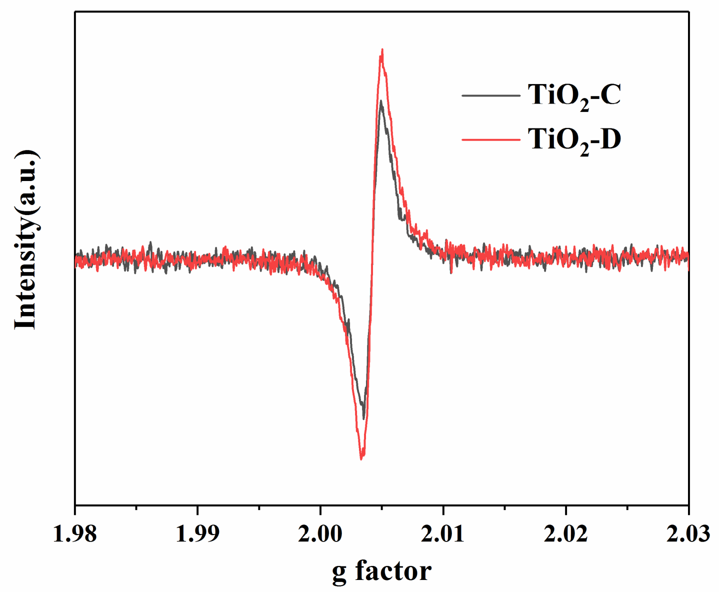

Figure S7. The EPR spectra of TiO_2-C and D samples.

S9. FTIR spectra, UV-vis DRS and XPS VB spectra of TCNX.

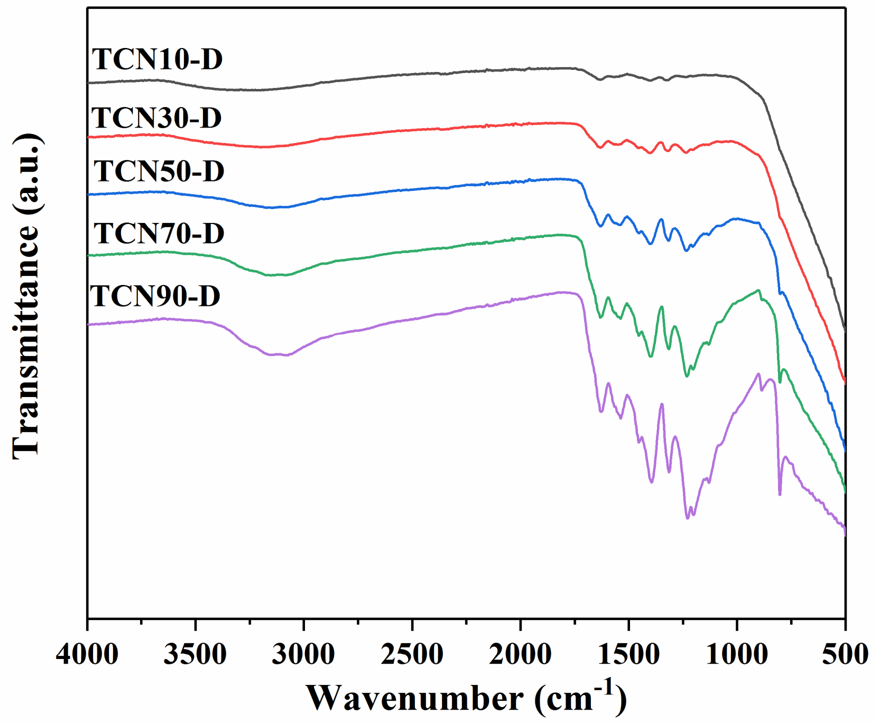

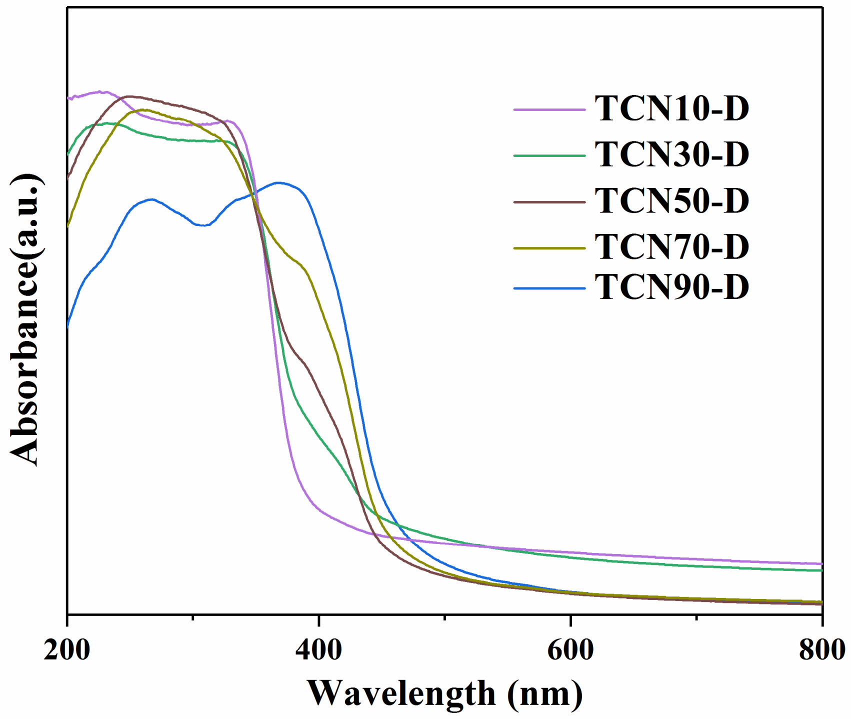

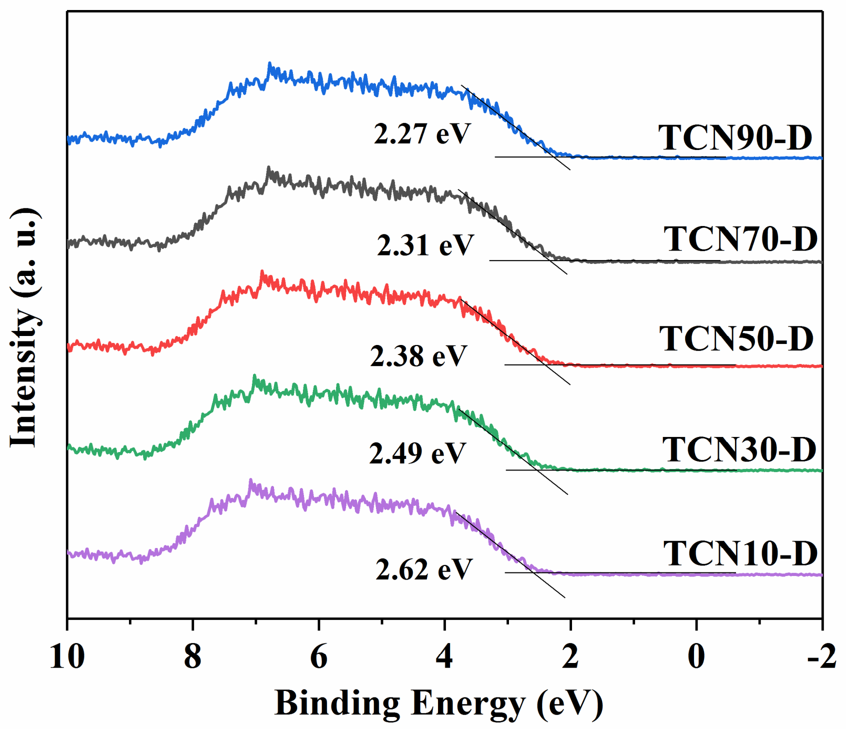

Figure S8. FTIR spectra, UV-vis DRS and XPS VB spectra of TCNX.

S10. EDS patterns and element mapping of TCN50-D sample

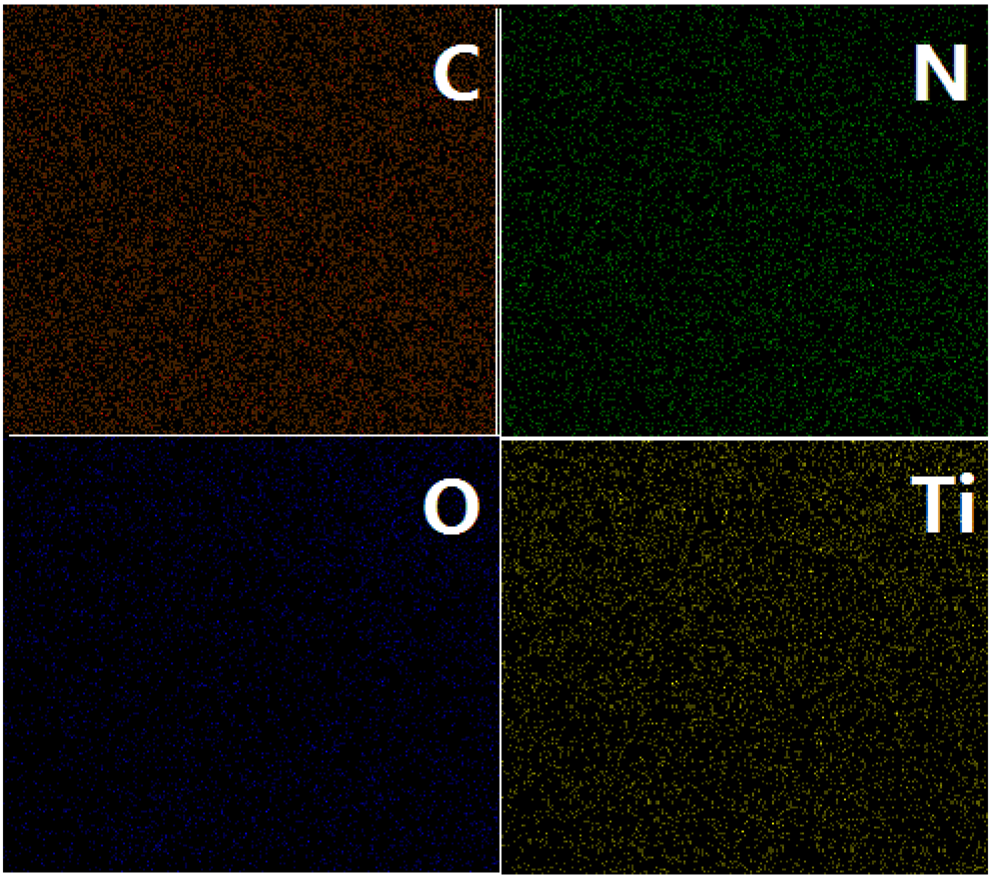

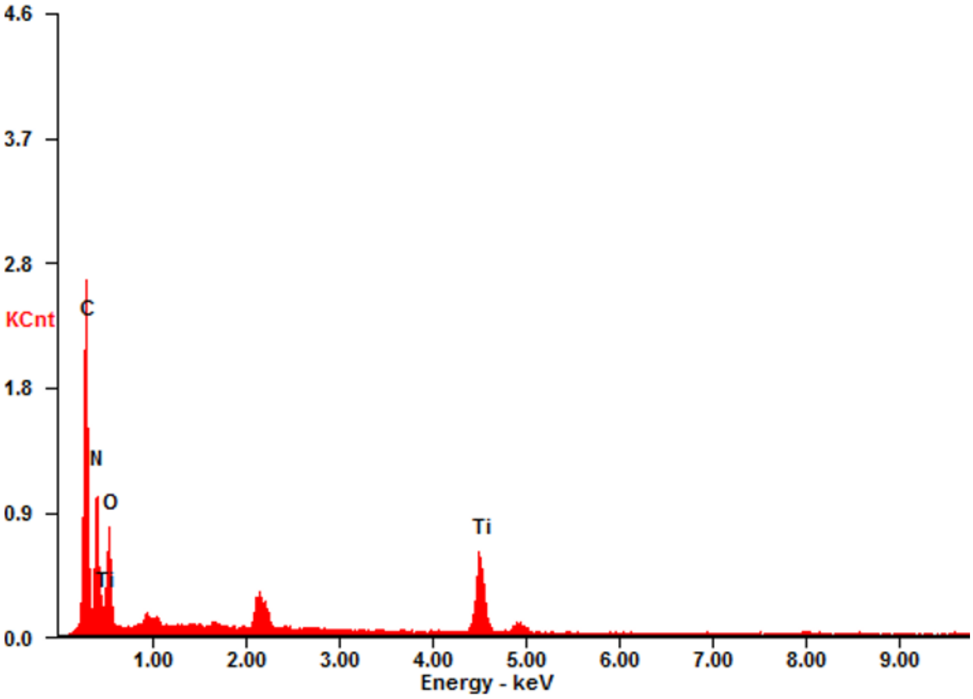

Figure S9. EDS patterns and element mapping of TCN50-D sample.

S11. Trapping experimental

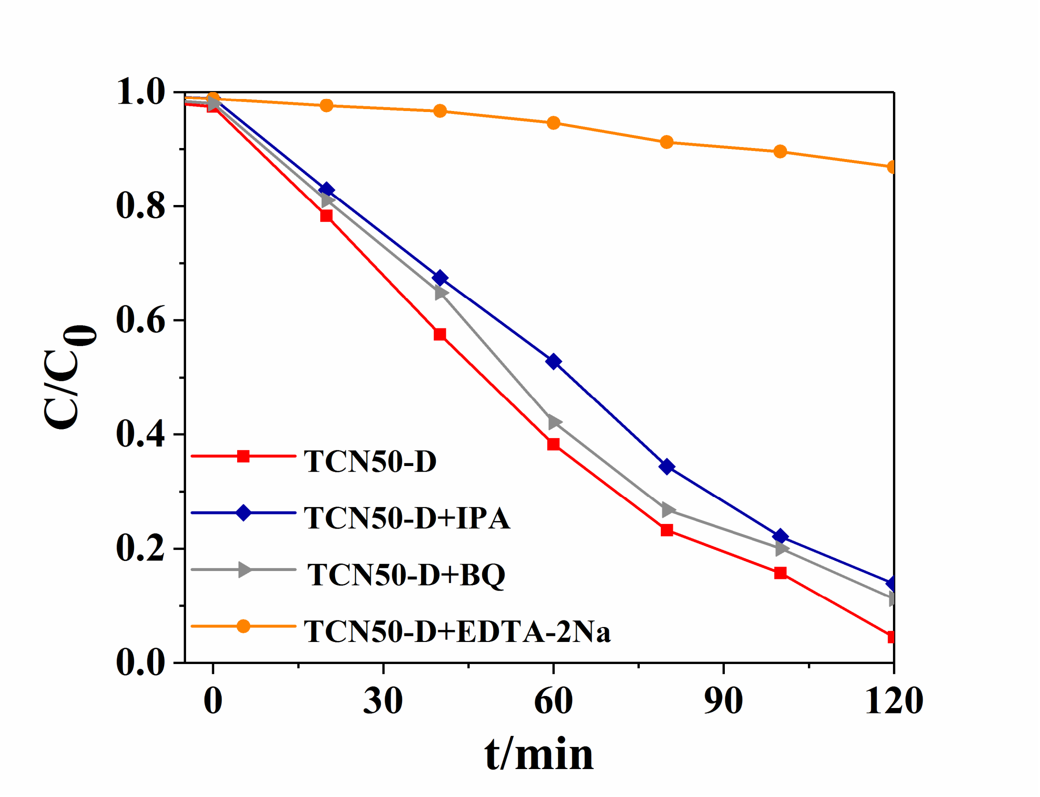

Figure S10. Trapping experimental of photogenerated radicals and holes in TCN50-D sample for the RhB degradation.

The photogenerated active substance trapping experiments was to determine the active substance. Before the start of the experiment of RhB degradation, a certain amount of benzoquinone, 2-propanol and ethylenediaminetetraacetic acid disodium salt were added to the mixture, corresponding to the scavenger of superoxide radicals, hydroxyl radicals and photogenerated holes, respectively.

S12. Valence band of XPS

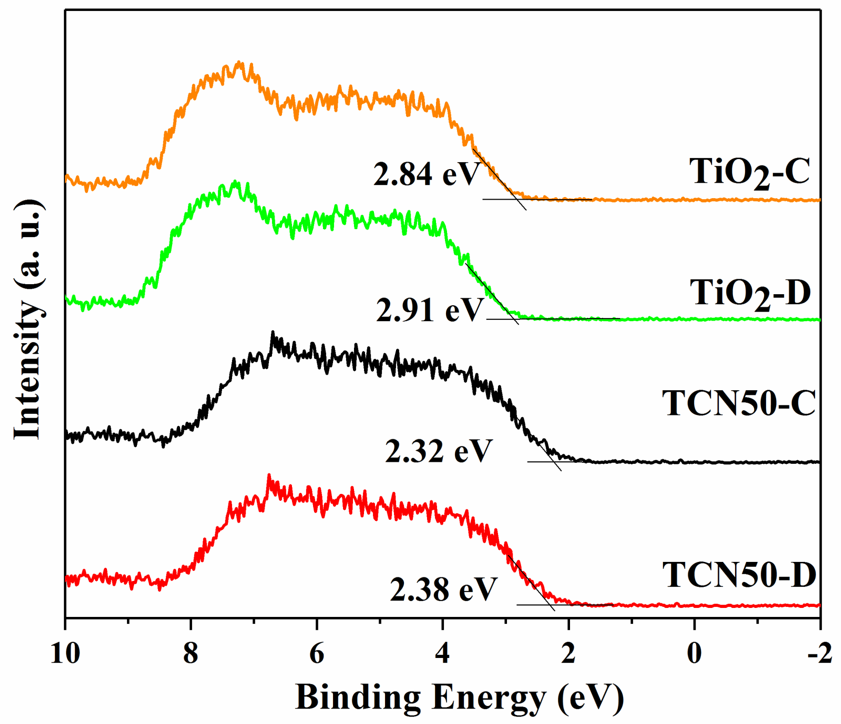

Figure S11. VB XPS of TiO_2-C, TiO_2-D, TCN50-C and TCN50-D.

Reference

[1] Ma, J.; Tan, X.; Yu, T.; Li, X. Fabrication of g-C_3N_4/TiO_2 hierarchical spheres with reactive {001} TiO2 crystal facets and its visible-light photocatalytic activity. *Int. J. Hydrogen Energ*. 2016, *41*, 3877-3887.

By measuring the intersection of the slope of the XPS valence band curve and the X axis, the valence band (VB) of the material can be determined.
